# 

**Published:** 1880-03

**Authors:** 


### Table of Contents.

### ORIGINAL COMMUNICATIONS.

The Bandage as Used by Professor Dudley and his Proselytes. By F. L. Merriwether, Houston, Texas........................ 707
Valedictory Address to the Graduating Class of the Atlanta Medical College of 1880. By J. A. Hurt, M D., Hurtville, Ala. 715

### REPORT OF SOCIETIES.

St. Louis Obstetrical and Gynecological Society........................... 720
Medical and Surgical Society of Baltimore.................................. 733

### SELECTIONS.

Spontaneous Cure of Organic Disease of the Heart........................ 738
Case of Spontaneous Rupture of the Uterus, with Recovery............ 748
Aspirator in Retention of Urine............................................... 751
Glioma of Retina.......................................................................... 754

### EDITORIAL.

National Board of Health........................................................... 757
Hydrate of Chloral.................................................................... 761
Meteorological Report for February......................................... 762

### BIBLIOGRAPHICAL.

The Microscope and Microscopical Technology............................ 763
A Practical Treatise on Nervous Exhaustion............................... 763

### EXCERPTA.

Chloroform .................................................................................. 764
Surgical Treatment of Goitre...................................................... 765
A Physiological Curiosity............................................................ 766
Ixdex to Volume XVII................................................................ 767

